# Association between genotype and disease complications in Egyptian patients with beta thalassemia: A Cross-sectional study

**DOI:** 10.1038/s41598-018-36175-9

**Published:** 2018-12-07

**Authors:** Tamer Hassan, Marwa Zakaria, Manar Fathy, Mohamed Arafa, Sherif El Gebaly, Ahmed Emam, Attia Abdel Wahab, Mohamed Shehab, Hosam Salah, Mai Malek, Khaled El Gerby

**Affiliations:** 1 Pediatric, Zagazig University, Zagazig, Egypt; 2 Clinical pathology, Zagazig University, Zagazig, Egypt; 3 Microbiology, Zagazig University, Zagazig, Egypt; 4 Radiodiagnosis departments, Zagazig University, Zagazig, Egypt

## Abstract

In beta thalassemia, the degree of globin chain imbalance is determined by the nature of the mutation of the β-gene. β° refers to the complete absence of production of β-globin on the affected allele. β^+^ refers to alleles with some residual production of β-globin. The homozygous state results in severe anemia that necessitates regular blood transfusion. On the other hand, frequent blood transfusion can lead to iron overload resulting in progressive dysfunction of the heart, Liver as well as multiple endocrinopathies. We studied the impact of genotype on the development of disease complications in patients with β thalassemia. A Cross sectional study was carried on 73 patients with beta thalassemia. Genotyping was determined by DNA sequencing technique. Routine investigations as well as MRI liver and heart were performed to assess iron overload. We found that β^+^β^+^ was the most common genotype in our patients followed by β°β° and β°β^+^. Mean Liver iron content (LIC) was significantly higher in β°β° compared to β°β^+^ and β^+^β^+^ genotypes and mean cardiac T2* was significantly lower in β°β° compared to β°β^+^ and β^+^β^+^ genotypes. Hepatic complications, hepatitis C, cardiac complications and some endocrinopathies were significantly higher in patients with β°β° genotype compared to other genotypes which explain the role of the underlying genetic defect in thalassemia patients in development of disease complications.

## Introduction

β-Thalassemias are inherited hemoglobin disorders which represent major health problem in Mediterranean countries^1^.

The clinical severity of β-thalassemia varies widely ranging from asymptomatic to severe or even fatal entities which reflects the degree of imbalance between α- and non-α-globin chains^2^. More than 300 disease-causing mutations have been identified. The broad spectrum of β-thalassemia alleles can produce a wide spectrum of diferent β- thalassemia phenotypes^3^.

Homozygous β-thalassemia is characterized by severe anemia and transfusion dependency^4^. Excess iron deposition in many tissues as a result of chronic transfusion, increased gastrointestinal iron absorption as a consequence of ineffective erythropoiesis, in addition to the hepcidin–ferroportin axis malfunctions results in organ damage, significant morbidity as well as significant mortality in thalassemia patients especially if untreated^5^.

Hepatic and cardiac dysfunctions as well as multiple endocrine abnormalities are the most encountered disease complications due to excessive iron overload^6^. Many previous data suggested that the clinical severity of the disease and development of such complications can be influenced by the type of β-thalassemia mutation and genotype^7^.

Our aim is to study the association between genotype and phenotype in patients with β-thalassemia.

## Results

### Demographic, transfusion and chelation characteristics of patients

The mean age of our patients was 13.8 years. They were 39 males (53.4%) and 34 females (46.6%). The mean age of start transfusion was 10 months and the mean transfusion frequency was 4.1 weeks. The mean age of start chelation was 3.1 years. Deferasirox was the most commonly used iron chelator followed by Deferiprone and Desferrioxamine (63.9%, 18% and 11.5% respectively). The mean compliance was 80.8% and the mean serum ferritin was 3385.8 ng/ml (Table 1).

**Table 1:** Clinical characteristics of our patients.

Demographic Data	N = 73
**Age (years)**	
Mean ± SD (Range)	13.8 ± 3.9 (6–23)
**Gender (n, %)**	
Males	39 (53.4)
Females	34 (46.6)
**Transfusion Data**	
Mean ± SD (Range)	
**Age of start (months)**	10.0 ± 11.7 (3–84)
**Frequency (weeks)**	4.1 ± 3.7 (2–24)
**Chelation Data**	
**Age of start (years)**	
Mean ± SD (Range)	3.1 ± 1.4 (2–8)
**Type (n, %)**	**N** **=** **61**
DFX	39 (63.9)
DFP	11 (18)
DFO	7 (11.5)
DFO + DFX	2 (3.3)
DFO + DFP	2 (3.3)
**Compliance (%)**	
Mean ± SD (Range)	80.8 ± 22.9 (25–100)
**Serum Ferritin (ng/ml)**	
Mean ± SD (Range)	3385.8 ± 1968.9 (900–7430)

SD: Standard deviation; DFX: Deferasirox; DFP: Deferiprone; DFO: Desferrioxamine; ng: nanogram; ml: milliliter.

### Clinical characteristics and disease complications of patients

Hepatomegaly was present in 41 patients (56.2%). 19 (26%) patients had hepatitis C. 16 patients (21.9%) had cardiac complications. Splenectomy was performed in 14 patients (19.2%). Growth retardation and hypogonadism were the commonest endocrinal complications (68.5% and 49.3% respectively).

### Mutations and genotypes of patients

IVS 1–1, IVS 1–110 and IVS 1–6 were the commonest mutation in our patients (26.7%, 22.6% and 18.5% respectively) (Table 2).

**Table 2:** Frequency of different mutations in patients.

Mutation	N = 146	%
IVS 1–1	39	26.7
IVS 1–110	33	22.6
IVS 1–6	27	18.5
IVS 11–745	14	9.6
Codon 39	13	8.9
Codon 5	5	3.4
Promoter 87	4	2.7
Codon 15	4	2.7
IVS 11–848	3	2.0
Codon 37	2	1.4
Codon 44	1	0.7
Codon 27	1	0.7

β^+^β^+^ was the most common genotype followed by β°β° and β°β^+^ (49.3%, 37.0% and 13.7% respectively).

Homozygous IVS 1–1, homozygous IVS 1–110 and homozygous IVS 1–6 were the commonest genotypes (19.17%, 15.06% and 10.95% respectively) (Table 3).

**Table 3:** Frequency of different genotypes (based on type of mutation) in our patients and their relationship with clinical diagnosis.

Genotype	N = 73 (N, %)	Clinical diagnosis
IVS 1–1 - IVS 1–1	14 (19.17)	Thalassemia Major
IVS 1–110 - IVS 1–110	11 (15.06)	Thalassemia Major
IVS 1–6 - IVS 1–6	8 (10.95)	Thalassemia Intermedia (6 patients) Thalassemia Major (2 patients)
IVS 1–1 - IVS 1–110	4 (5.47)	Thalassemia Major
IVS 11–745 - IVS 11–745	4 (5.47)	Thalassemia Major
IVS 1–1 - Codon 39	4 (5.47)	Thalassemia Major
IVS 1–110 - IVS 11–745	3 (4.1)	Thalassemia Major
Codon 39 - Codon 39	3 (4.1)	Thalassemia Major
IVS 1–110 - IVS 1–6	2 (2.73)	Thalassemia Major
IVS 1–1 - IVS 11–745	2 (2.73)	Thalassemia Major
IVS 1–6 - Codon 39	2 (2.73)	Thalassemia Major
Codon 15 - Codon 15	2 (2.73)	Thalassemia Major
IVS 1–6 - Promoter 87	2 (2.73)	Thalassemia Intermedia
IVS 1–6 - IVS 1–5	2 (2.73)	Thalassemia Intermedia
IVS 1–1 - IVS 1–6	1 (1.36)	Thalassemia Major
IVS 1–110 - Codon 39	1 (1.36)	Thalassemia Major
Codon 44 - Codon 27	1 (1.36)	Thalassemia Major
IVS 11–848 - IVS 11–848	1 (1.36)	Thalassemia Intermedia
IVS 1–6 - IVS 11–848	1 (1.36)	Thalassemia Major
IVS 1–110 - Codon 5	1 (1.36)	Thalassemia Major
Codon 37 - Codon 37	1 (1.36)	Thalassemia Major
Promoter 87 - Promoter 87	1 (1.36)	Thalassemia Intermedia
IVS 1–6 - IVS 11–745	1 (1.36)	Thalassemia Major
Codon 5 - Codon 5	1 (1.36)	Thalassemia Major

63% of our patients had the same mutations from both parents (homozygous) while 37% of our patients had two different mutations each from one parent (compound heterozygous). Parent consanguinity was present in 31.5%.

### Hepatic and myocardial iron content

The mean liver iron content was 17.4 mg/g dw (range: 1.1–37.2, median 16) and mean cardiac T2* was 25.5 ms (range: 11.8–43.9, median 26).

83.6% of our patients had transfusion dependent thalassemia (thalassemia major) and 16.4% had non transfusion dependent thalassemia (thalassemia intermedia).

### Genotype- phenotype relationship

Patients of the three genotypes were matched as regards age and sex. Patients with β°β° received transfusion and chelation at an earlier time and they received transfusion more frequently as opposed to those with β°β^+^ and β^+^β^+^ genotypes. 33.3% of patients with β^+^β^+^ genotypes did not receive iron chelation at all. Also, Patients with β°β° had poorer compliance and higher levels of serum ferritin in comparison to those with β°β^+^ and β^+^β^+^ genotypes (Table 4).

**Table 4:** Relationship between genotype and demographic, transfusion, chelation, clinical characteristics and disease complications in patients.

	β°β°(n = 27)	β°β^+^ (n = 10)	β^+^β^+^ (n = 36)	Test	P
**Demographic data**					
Age (years)					
Mean ± SD (range)	14.5 ± 4.3 (8–23)	16.2 ± 4.4 (11–23)	14.0 ± 3.1 (6–22)	F = 1.2	0.29
Gender (n, %)					
Male	15 (55.6)	5 (50)	19 (52.8)	X^2^ = 0.2	0.9
Female	12 (44.4)	5 (50)	17 (47.2)		
**Transfusion data**					
Age of start (months)					
Mean ± SD (range)	4.8 ± 2.1 (2–9)	6.6 ± 3.6 (3–12)	16.6 ± 15.8 (2–84)	Kw = 56.4	<0.001
Frequency(weeks)					
Mean ± SD (range)	2.5 ± 1.0 (2–7)	2.8 ± 0.7 (2–4)	6 ± 4.9(2–24)	Kw = 64.9	<0.001
**Chelation data**					
Age of start(years)					
Mean ± SD (range)	2.5 ± 1 (2–7)	2.7 ± 0.9 (2–5)	4 ± 1.3 (2–8)	F = 21	<0.001
**Chelator type (n, %)**					
DFX	17 (63)	5 (50)	17 (47.2)	X^2^ = 17.06	0.009
DFO	5 (18.5)	1 (10)	1 (2.8)		
DFP	2 (7.4)	3 (30)	6 (16.7)		
Combined	3 (11.1)	1 (10)	0 (0.0)		
No chelator	0 (0.0)	0 (0.0)	12 (33.3)		
**Compliance (%)**					
Mean ± SD (range)	69.6 ± 26 (25–100)	85 ± 17 (50–100)	91.7 ± 14 (50–100)	F = 14.9	<0.001
**Serum Ferritin (ng/ml)**					
Mean ± SD (range)	4958 ± 1416 (1745–7430)	3158 ± 1761 (1300–7000)	1345 ± 1004 (280–5000)	F = 121.9	<0.001
**Hepatomegaly (n, %)**	22 (81.5)	6 (60)	13 (36.1)	X^2^ = 25.9	<0.001
**Hepatitis C (n, %)**	11 (40.7)	4 (40)	4 (11.1)	X^2^ = 16.4	<0.001
**Cardiac (n, %)**	12 (44.4)	2 (20)	2 (5.6)	X^2^ = 27.3	<0.001
**Splenectomy (n, %)**					
Yes	11 (40.7)	2 (20)	1 (2.8)	X^2^ = 14.35	<0.001
No	16 (59.3)	8 (80)	35 (97.2)		

SD: Standard deviation; DFX: Deferasirox; DFP: Deferiprone; DFO: Desferrioxamine; ng: nanogram; ml: millilitre.

Hepatomegaly and hepatitis C were more prevalent in β°β° as opposed to those with β°β^+^ and β^+^β^+^ genotypes (81.5%, 60% and 36.1% respectively for hepatomegaly and 40.7%, 40%, 11.1% respectively for Hepatitis C). Patients with β°β° were associated with greater prevalence of cardiac complications, growth retardation, hypogonadism, hypothyroidism compared to those with β°β^+^ and β^+^β^+^ genotypes (44.4%, 20% and 5.6% respectively for cardiac complications, 96.3%, 90%, 41.7% respectively for growth retardation, 81.5%, 60.0% and 22.2% respectively for hypogonadism and 18.5%, 0.0% and 5.6% respectively for hypothyroidism). Splenectomy was significantly higher in patients with β°β° compared to patients with β°β^+^ and β^+^β^+^ (40.7%, 20%, 2.8% respectively) (Table 4).

Patients with β°β° were associated with significantly higher liver and myocardial iron content compared to those with β°β^+^ and β^+^β^+^ genotypes where mean LIC was significantly higher in β°β° compared to β°β^+^ and β^+^β^+^ genotypes (27.3, 12 and 6.4 mg/g dw respectively) and mean cardiac T2* was significantly lower in β°β° compared to β°β^+^ and β^+^β^+^ genotypes (21.3, 26.6 and 33.1 ms respectively) (Table 5).

**Table 5:** Relationship between genotype and liver iron content and cardiac T2* in our patients.

	β°β° (N = 27)	β°β^+^ (N = 10)	β^+^β^+^ (N = 36)	F	P
**LIC (mg/g dw)**					
Mean ± SD	27.3 ± 6.7	12 ± 7.9	6.4 ± 4.5	96.09	<0.001
Range	13.4–37.2	1.1–27.2	1.1–16.9		
T2* (ms)					
Mean ± SD	21.3 ± 7.7	26.6 ± 6.1	33.1 ± 7.1	40.8	<0.001
Range	11.8–43.9	18.2–40.4	13.4–43.6		

All patients with thalassemia intermedia belong to β^+^β^+^, while all patients with β°β° presented with transfusion dependent thalassemia major.

### Relationship between Serum ferritin and demographic, transfusion, chelation characteristics and disease complications

**Figure 1:**
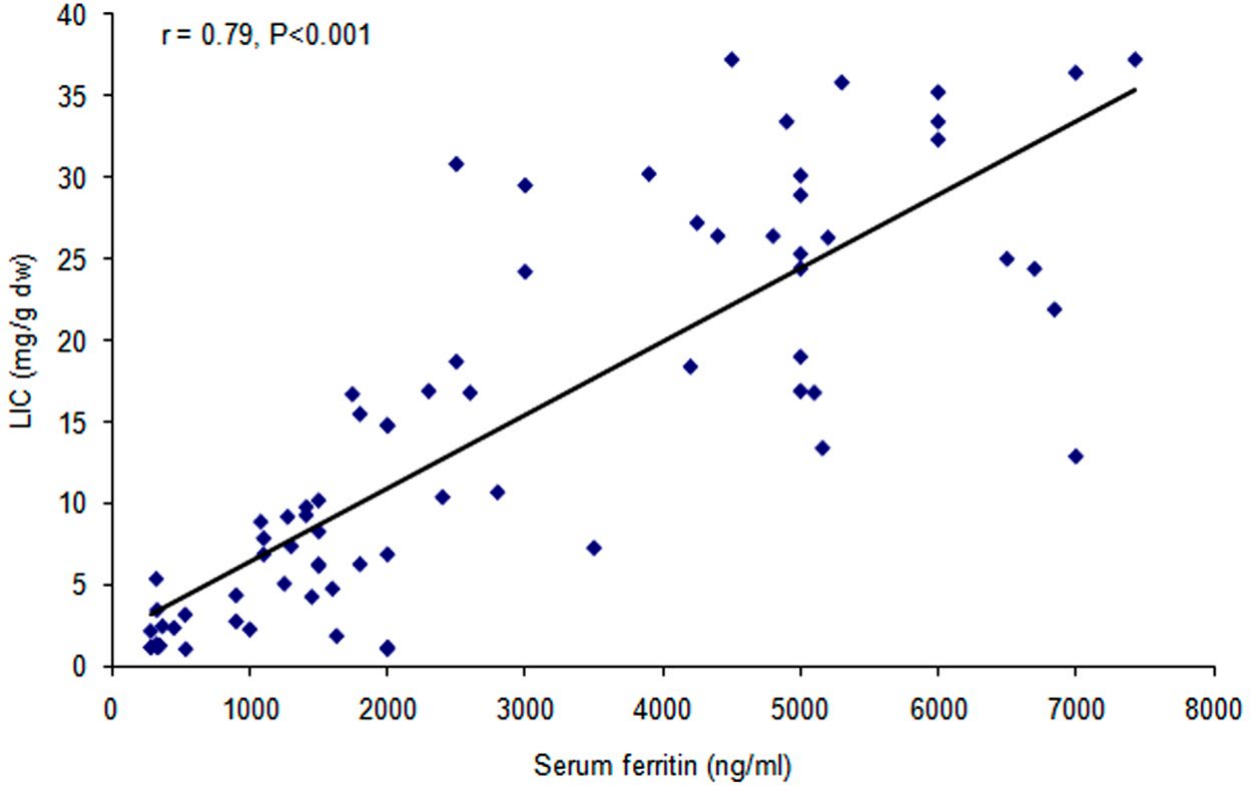
Correlation between serum ferritin and LIC. There was highly significant positive correlation between serum ferritin and LIC.

**Figure 2:**
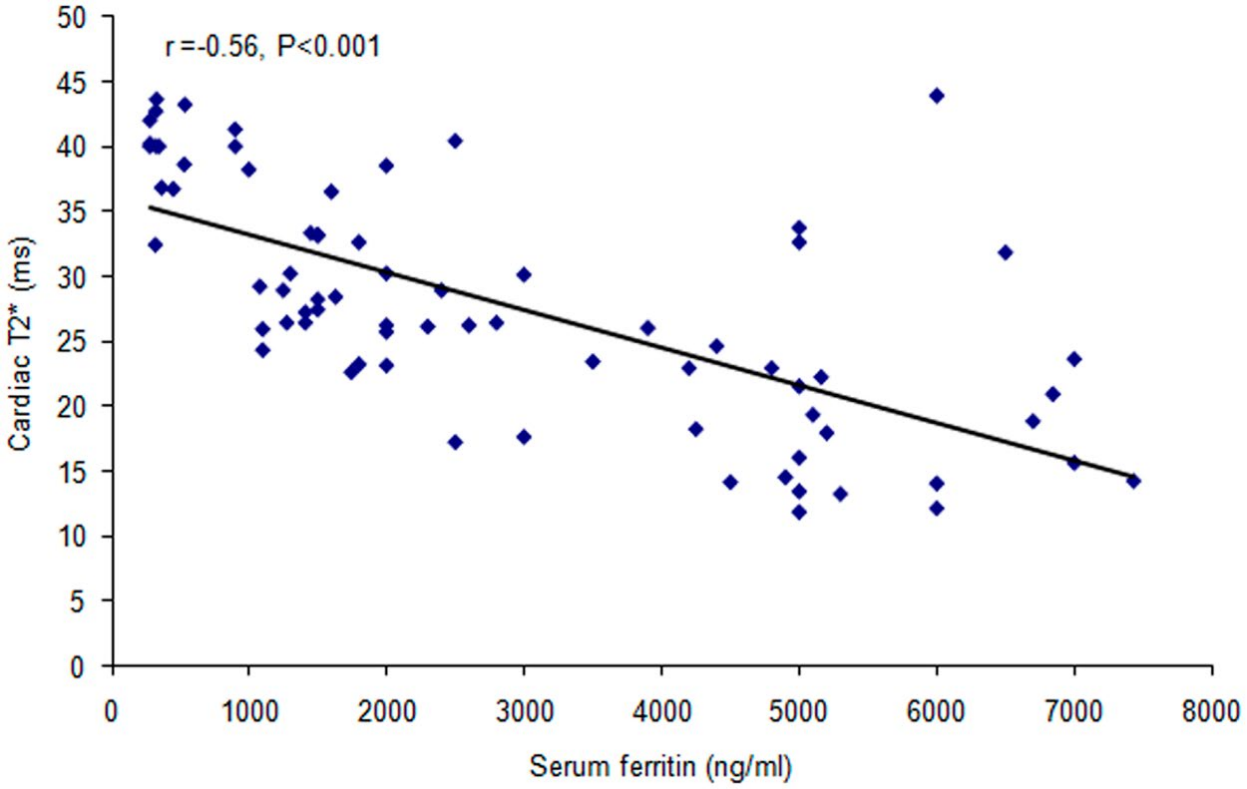
Correlation between serum ferritin and cardiac T2*. There was highly significant negative correlation between serum ferritin and cardiac T2*.

There was highly significant positive correlation between serum ferritin and each of age (r = 0.43, p < 0.001) and liver iron content (r = 0.79, p < 0.001) and highly significant negative correlation between serum ferritin and each of age of start transfusion (r = −0.56, p < 0.001), frequency of transfusion (r = −0.58, p < 0.001), age of start chelation (r = −0.52, p < 0.001), compliance (r = −0.52, p < 0.001) and cardiac T2* (r = −0.56, p < 0.001) (Figs 1 and 2).

## Discussion

Although the clinical management of beta thalassemia has changed dramatically, yet there is a wide spectrum of complications which arise mainly from obligatory lifelong blood transfusion and the subsequent iron overload^8^. Hepatic diseases remain one of the most important problems among patients with thalassemia. Common causes include transfusion-related viral hepatitis (Hepatitis B, C), iron overload, drug toxicity, and biliary disease due to gallstones^9^. Cardiac failure and rhythm disturbances remain the main cause of death among young adults with beta thalassemia major^10^.

Growth retardation, Hypogonadism, hypothyroidism, diabetes mellitus, low bone mass, and hypoparathyroidism are still the most common encounterted endocrine problems in young adults with thalasemia major^11^. Previous data suggest that beta thalassemia genotype which determines the disease severity, can also be a key contributing factor in development of these complications and due to broad spectrum of β-thalassemia alleles different β-thalassemia phenotypes can result^12^.

In our study, Hepatomegaly was present in 41 patients (56.2%). 19 (26%) of patients had hepatitis C. 16 patients (21.9%) of our patients had cardiac complications. As regards endocrinal complications, growth retardation and hypogonadism were the commonest ones (68.5% and 49.3% respectively) followed by hypoparathyroidism, diabetes mellitus and hypothyroidism (17.8%, 15.1% and 9.6% respectively).

As for prevalence of hepatomegaly in thalassemic patients, variable results were obtained due to differences in studied populations and age of included patients. Hashemizadeh *et al*., found hepatomegaly in 46% of beta thalassemia major patients with a mean age of 10.8 years^13^. Higher percentage (77%) of hepatomegaly was reported by Caocci *et al*. in thalassemic patients with a median age of 10 years^14^. On the contrary, Grow *et al*., reported lower prevalence of hepatic complications (6.8%) and this can be explained based on that 59.3% of their patients were below 10 years^15^.

Cardiac complications were present in 21.9% of our patients and this is nearly consistent with Grow *et al*. who found cardiac complications in 23.3% of thalassemic patients^15^. However, Ansari *et al*. reported lower prevalence of cardiac complications (13.2%) in their patients^16^.

Growth retardation was the commonest disease complication in our study (68.5%). This was supported by many other studies with a range from 49.4% to 90.9%^15,17,18^. Growth retardation in thalassemic patients can be attributed to growth hormone neuro-secretary disturbance and secondary growth hormone insensitivity, chronic anemia, congestive cardiac failure, haemosiderosis and other endocrine and metabolic disturbances may also be contributory factors^19^.

Hypogonadism was reported in 49.3% of our patients. Higher percentage (54.7% to 69%) was found in some studies^17,20,21^ while, lower prevalence (12.2 to 32.5%) was observed by others^19,22^.

Diabetes mellitus (DM) was found in 15.1% of our patients. This finding is quite near to Ansari *et al*., who found that the prevalence of DM was 13.2%^16^. Our results were higher than some studies (8% to 10%)^17,22,23^ and lower than was reported by Jensen *et al*. (20%). Higher prevalence of DM reported by Jensen *et al*., was closely tied to genotype and age of patients^24^.

Hypoparathyroidism was present in 17.8% of our patients. Jensen *et al*. and Gulati *et al*. found hypoparathyroidism in 13% of patients^24,25^. Lower prevalence of hypoparathyroidism was reported by Toumba *et al*. (1.2%)^22^ and Al Akhras *et al*. (7%)^17^.

As for hypothyroidism, it was present in 9.6% of our patients. This finding goes in line with Al Akhras *et al*., and Shamshirsaz *et al*., where the prevalence of hypothyroidism was 8% and 7.7% respectively^17,19^. Some studies reported higher prevalence of hypothyroidism (17–18%)^26–28^, while others found low prevalence (0 to 9%)^29,30^.

Variable results were imputable to differences in studied populations and age of patients.

In our study, IVS 1–1[G > A], IVS 1–110[G > A] and IVS 1–6[T > C] (26.7%, 22.6% and 18.5%, respectively) were the commonest mutations. These results were matched with many previous Egyptian studies. Though IVS 1–1[G > A], IVS 1–110[G > A] and IVS 1–6[T > C] were the commonest mutations in most Egyptian studies yet the order of frequency of these mutations is different in these studies^17,31–36^.

The discrepancy in the order of frequency of these mutations can be attributed to different study populations and different sample sizes.

As for Mediterranean populations, Huisman *et al*., found that the commonest mutations were IVS 1–110[G > A], IVS 1–6[T > C], IVS 1–1[G > A], promotor 87[C > G], IVS 11–745[C > G] and C39[C > T]. In addition, C8 [-AA], C8,/C9[+G], IVS 1–5[G > C], C39[C > T], C44[-C] and IVS 11–828[C > A] were the most prevalent mutations in Middle East areas^37^.

In our study, 63% of our patients had the same mutations from both parents (homozygous) while 37% of our patients had two different mutations each from one parent (compound heterozygous). Homozygous IVS 1–1[G > A], homozygous IVS 1–110[G > A] and homozygous IVS 1–6[T > C] were the commonest genotypes in our patients (19.17%, 15.06% and 10.95% respectively).

Elmezayen *et al*., found homozygous mutations in 51% and compound heterozygous mutations in 28% of patients and reported that the most common genotypes were homozygous IVS 1–6[T > C], IVS 1–110[G > A], promotor 87[C > G], IVS 1–6[T > C]/IVS 1–110[G > A] and IVS 1–6[T > C]/IVS 11–848[C > A] (15%, 13%, 6%, 6% and 6%)^36^.

On the contrary, El-Shanshory *et al*. found compound heterozygous mutations to be more common than homozygous mutations^32^. Higher percentage of homozygous mutations (63%) in our study can be attributed to the higher consanguineous marriage (31.5%) in our study populations.

In our study, the mean liver iron content was 17.4 mg/g dw and mean cardiac T2* was 25.5 ms. Pathological cardiac T2* values (≤20 ms) were found in 16 patients (21.9%).

Our data are matched with those reported by Wood *et al*., where the mean hepatic iron concentration was 18.4 ± 3.8 4 mg/g dw and cardiac T2* was 26.1 ± 4.6 ms^38^. Also our results go in line with Eghbali *et al*., where they found the mean cardiac T2* was 26.46 ± 9.19 ms^39^. Similarly, Fragasso *et al*., found the mean cardiac T2* was 27 ± 15 ms, pathologic values (≤20 ms) were found in 37 (37%) patients in the thalassemia major cohort and mean cardiac T2* were 30 ± 11 ms, pathologic values in 3 (15%) patients in the thalassemia intermedia cohort^40^.

A large Phase II randomized study (CORDELIA study) included 925 patients with transfusion dependent anemia from 11 countries of which 387 patients were from Egypt, revealed higher mean LIC (25.8 mg/g dw) compared to our results. Lower mean cardiac T2* values (21.8 ms) were also reported in CORDELIA study than ours. Also, higher percentage (36.7%) of pathological cardiac T2* (≤20 ms) were observed in CORDELIA study compared to ours^41^. Lower LIC and higher cardiac T2* in our study compared to CORDELIA study can be attributed to younger age of our patients than CORDELIA study (13.8 versus 19.8 years respectively). Also CORDELIA study included patients other than beta thalassemia (Diamond Blackfan anemia, myelodysplasia and sideroblastic anemia).

On the contrary, Verlhac *et al*. found that median LIC was 6.15 mg/g dw in their study^42^. Lower LIC in this study in comparison to our study can be explained by younger age of their patients (9.9 versus 13.8 years in our study). Also, Verlhac included only patients with sickle cell disease with lower transfusion demands and subsequent lower iron loading.

In our study, patients with β°β° received transfusion and chelation at an earlier time and they received transfusion more frequently as opposed to those with β°β^+^ and β^+^β^+^ genotypes. 33.3% of patients with β^+^β^+^ genotypes did not receive iron chelation at all. Also, Patients with β°β° had poorer compliance and higher levels of serum ferritin in comparison to those with β°β^+^ and β^+^β^+^ genotypes.

Consistent with our findings, Al Akhras *et al*. found that patients with the β°β° genotype had more severe phenotype and this was evident in the younger age of start transfusion and chelation, as well as the more frequent transfusions in these patients as opposed to patients with other genotypes^17^. This was also supported by Chern *et al*.^7^ and Skordis *et al*.^43^.

In our study, disease complications were significantly higher in patients with β°β° genotype. Our results were matched with those reported by Yaman *et al*., who found that the rate of complications was significantly evident (P < 0.05) in patients with β°β° genotype compared to thalassemia intermedia patients with β^+^β^+^^44^.

Also, Winichagoon *et al*., in their study on 144 patients with beta-thalassemia were divided into mild (46 patients), intermediate (55 patients), and severe groups (43 patients) reported that Two alleles of mild beta-thalassemia mutation β^+^β^+^ thalassemia or β+/Hb E) resulted in a mild clinical symptom whereas two alleles of severe beta-thalassemia mutation β°β°produced a severe clinical phenotype^45^. This was also supported by Kattamis and his colleagues^46^.

Mutations in beta globin gene impact hepatic and myocardial iron overload as well as other disease complications through their effect on the degree of imbalance between the α- and non-α-globin chains. β°β° genotype is associated with higher degree of imbalance between the α- and non-α-globin chains resulting in higher rate of hemolysis and increased transfusion frequency with concomitant higher iron overload in liver and heart. Genotype can also influence phenotype through the age at which patient started iron chelators with its deleterious side effects^47^.

Regarding genotypes of patients with thalassemia intermedia in our study, homozygous IVS 1–6 accounted for 50% of patients genotypes and compound heterozygous IVS 1–6 with other mutations accounted for 33.3% of patients genotypes while homozygous promotor 87 and homozygous IVS 11–848 accounted for 16.7% of patients genotypes. These data should shed light on IVS 1–6 as a benign mutation.

These results are concordant with that observed by Shamoon *et al*. where IVS-I-6 (T > C) was the most frequently encountered mutation (34.6%) in patients with thalassemia intermedia^48^.

Our results showed that all patients with thalassemia intermedia belong to β^+^β^+^ genotype while all patients with β°β° genotype presented with transfusion dependent thalassemia major.

Camaschella *et al*., in their study on 292 Italian patients, 165 with thalassemia intermedia and 127 with thalassemia major reported that homozygousity for mild mutations β^+^β^+^ accounts for 24% of the intermedia patients and it is not represented among major patients. Forty-four percent of intermedia patients had combinations of mild/severe β°β^+^mutations and 32% had homozygousity or double heterozygosity for severe mutations β°β°, Seventy-six percent of patients with thalassemia major were classified in β°β° and 24% in β°β^+^^49^.

Limitations in this study were small sample size and patients were from limited geographical areas and not representing whole Egypt. Higher study costs was the main reason for our small sample size. Larger multicenter studies are still needed to support these findings.

## Materials and Methods

A cross sectional study was carried on 73 thalassemia patients (39 males, 34 females) aged over 10 years during their follow up visits to hematology outpatient clinic of Zagazig university hospital. Data abstraction form was designed to capture appropriate medical information from patients records.

### Inclusion criteria

- Patients with beta thalassemia whether major or intermedia.
- Age: ≥6 years old.
- Sex: both sex.
- Approval to participate in the study and signing the required assent and/or consent forms.

### Exclusion criteria

- Patients with chronic hemolytic anemias other than Beta thalassemia.
- Age: <6 years old.
- Refusal to participate in the study.

### All patients were subjected to the following

- Full medical history and thorough physical examination.
- Assessment of anthropometric measures.
- Tanner score for assessment of pubertal status.
- Menstrual history in girls.
- Routine investigations according to our local standards e.g. CBC, Serum ferritin,Liver function tests, Kidney function tests, hormonal profile [growth hormone basal and after provocation, TSH, free T4, LH, FSH, parathormone, estradiol (in girls) and testosterone (in boys)],echocardiography, etc.
- Genotyping was done using DNA sequencing technique in hemoglobinopathies laboratory of Ulm University, Ulm, Germany. For DNA analyses, polymerase chain reaction (PCR)-amplified complete sequencing of the beta-chain genes was performed. The PCR products were studied as single-strand DNA in comparison to normal control samples, and the type of anomaly was determined by comparison of the nucleic acid sequence of the altered DNA with that of normal DNA.
- MRI liver and heart to determine hepatic and myocardial iron overload using 1.5 Tesla MRI system (Philips Intera Achieva; Philips Medical Systems, Best, The Netherlands).

### Definitions

- Short stature was defined as height more than 2 SD below mean for age, sex and race^50^.
- Hypogonadotropic hypogonadism was defined as LH and FSH levels below 2 IU/L, with an estradiol concentration of below 20 pg/mL in girls or a testosterone concentration of below 3 ng/mL in boys^7^.
- Hypogonadism was defined by lack of breast development in girls and lack of testicular enlargement in boys(less than 4 ml) as measured by preorcidometer by the age of 16 year^51^.
- Hypothyroidism was defined as a low serum thyroxin (T4 < 4.5 µg/dl and T3 < 82 ng/dl) with an elevated serum TSH concentration (>4 µIU/ml)^52^.
- Hypoparathyroidism was defined as low parathormone level (with a reference range from 15–65 pg/ml), low total and ionized serum calcium, high serum phosphate, normal serum magnesium and alkaline phosphatase levels^19^.
- Diagnosis of DM was based on measurement of fasting blood glucose level according to American Diabetes Association, WHO Criteria and National Diabetes Health Group 1979^53^.
- Patients were considered to have cardiac complications if they have positive cardiac history (palpitations, irregular heart rate, chest pain, dyspnea, exercise intolerance, nocturnal cough, orthopnea, dependent edema, or unexplained fevers) and exam (systemic or pulmonary venous congestion, gallop, and edema) and/or abnormal echocardiographic or electrocardiographic findings.
- Patients were considered to have Hepatitis C based on a positive PCR result.
- Cardiac T2* above 20 ms was considered normal, 15–20 ms: mild cardiac iron overload, 10–15 ms: moderate cardiac iron overload and below 10 ms: severe cardiac iron overload. Mean liver iron content (LIC) below 3 mg/g dw was considered normal. 3–7 mg/g dw: mild liver iron overload, 7–14 mg/g dw: moderate liver iron overload and ≥14 mg/g dw: severe liver iron overload^54^.

### Criteria to start chelation therapy

Iron chelation therapy was started after the cumulative transfusion of 10 units of Packed RBCs or when serum ferritin was greater than 1,000 ng/mL^1^.

### Statistical analysis

SPSS version 20 (IBM SPSS, Armonk, NY, USA) was used for data analysis. Mean ± standard deviation was used for quantitative variables, while number and percentage were used for qualitative ones. X^2^ test, t-test, ANOVA, Kruskal Wallis, Mann Whitney and correlation coefficient tests were used when appropriate. P < 0.05 and P < 0.001 were considered to indicate significant and highly significant differences respectively.

### Statement of Ethics

The present study was performed according to Helsinki Declaration of 1964, as revised in 2000, and was approved by ethics committee of faculty of medicine, Zagazig University. Informed written consent and/or assent were obtained from all study participants and/or their care givers.

## Data Availability

The datasets generated during and/or analyzed during the current study are available from the corresponding author on reasonable request.

## References

[1] Galanello R, Origa R (2010). Beta thalassemia. Orphanet J Rare Dis..

[2] Olivieri NF (1999). The (beta)-thalassemias. N Engl J Med..

[3] Finotti A, Gambari R (2014). Recent trends for novel options in experimental biological therapy of β-thalassemia. Expert Opinion on Biological Therapy..

[4] Taher, A. T., Weatherall, D. J. & Cappellini, M. D. Thalassaemia. Lancet. pii: S0140-6736, 31822–31826 (2017).

[5] Nemeth E (2010). Hepcidin in beta-thalassemia. Ann N Y Acad Sci..

[6] Nichols-Vinueza DX, White MT, Powell AJ, Banka P, Neufeld EJ (2014). MRI guided iron assessment and oral chelator use improve iron status in thalassemia major patients. Am J Hematol..

[7] Chern JP (2003). Hypogonadotropic hypogonadism and hematologic phenotype in patients with transfusion dependent beta thalassemia. J Pediatr Hematol Oncol,.

[8] Old, J. M., Olivieri, N. F. & Thein, S. L. Management and prognosis. In: Weatherall, D. J., Clegg, J. B. eds *The Thalassaemia Syndromes*. London: Blackwell Science; 630–685 (2001).

[9] Brissot, P. & Cappellini, M.D. In: Liver disease. (Eds Cappellini, M. D., Cohen, A., Porter, J. *et al*.) Guidelines for the Management of Transfusion Dependent Thalassaemia (TDT). 3^rd^ ed. Thalassaemia International Federation, Ch 5, 143–159 (Nicosia (CY), 2014). 25610943

[10] Jensen PD (2003). Evaluation of myocardial iron by magnetic resonance imaging during iron chelation therapy with deferrioxamine: indication of close relation between myocardial iron content and chelatable iron pool. Blood.

[11] De Sanctis V (2002). Growth and puberty and its management in thalassaemia. Horm Res..

[12] Chen W (2010). The molecular basis of beta-thalassemia intermedia in southern China: genotypic heterogeneity and phenotypic diversity. BMC Med Genet..

[13] Hashemizadeh H, Noori R, kolagari SH (2012). Assessment Hepatomegaly and liver Enzymes in 100 Patients with beta Thalassemia Major in Mashhad, Iran. Iran J Ped Hematol Oncol..

[14] Caocci G (2012). Health related quality of life in Middle Eastern children with beta-thalassemia. BMC Blood Disorders..

[15] Grow K, Abrol P, Vashist M, Yadav R, Sharma S (2013). Associated Complications In Beta Thalassemia Patients. IOSR Journal of Pharmacy..

[16] Ansari SH (2014). Quality of life in patients with thalassemia major. Iran J Ped Hematol Oncol..

[17] Al-Akhras A (2016). Impact of genotype on endocrinal complications in β-thalassemia patients. Biomed Rep..

[18] Mostafavi H, Afkhamizadeh M, Rezvanfar MR (2005). Endocrine disorders in patients with thalassemia major. Iranian J Endocrinol Metab..

[19] Shamshirsaz AA (2003). Metabolic and endocrinologic complications in beta-thalassemia major: a multicenter study in Tehran. BMC Endocr Disord..

[20] Moayeri H, Oloomi Z (2006). Prevalence of growth and puberty failure with respect to growth hormone and gonadotropins secretion in beta-thalassemia major. Arch Iran Med..

[21] Pignatti C (2004). Survival and complications in patients with thalassemia major treated with transfusion and deferoxamine. Haematologica..

[22] Toumba M (2007). Endocrine complications in patients with thalassemia major. Pediatr Endocrinol Rev..

[23] Kurtoglu AU, Kurtoglu E, Temizkan AK (2012). Effect of iron overload on endocrinopathies in patients with beta-thalassaemia major and intermedia. Endokrynol Pol..

[24] Jensen CE (1997). Incidence of endocrine complications and clinical disease severity related to genotype analysis and iron overload in patients with beta-thalassaemia. Eur J Haematol..

[25] Gulati R, Bhatia V, Agarwal SS (2000). Early onset of endocrine abnormalities in beta-thalassemia major in a developing country. J Pediatr Endocrinol Metab..

[26] Soliman AT (2013). Longitudinal study on thyroid function in patients with thalassemia major: High incidence of central hypothyroidism by 18 years. Indian J Endocrinol Metab..

[27] Landau H (1993). Cross-sectional and longitudinal study of the pituitary-thyroid axis in patients with thalassaemia major. Clin Endocrinol (Oxf).

[28] Agarwal MB (1992). Thyroid dysfunction in multi-transfused iron loaded thalassemia patients. Indian Pediatr..

[29] Depaz G (1985). Thyroid function in thalassemia major. Ann Pediatr (Paris)..

[30] Senanayake MP, Suraweera SA, Hubert HD (1999). Thyroid function in thalassaemia major. Ceylon Med J..

[31] Soliman OE (2010). Reverse hybridization Strip Assay detection of beta-thalassemia mutations in northeast Egypt. Hematology..

[32] El-Shanshory M (2014). Spectrum of Beta Globin Gene Mutations in Egyptian Children with β-Thalassemia. Mediterr J Hematol Infect Dis..

[33] Hussein G (2007). Rapid detection of beta-Thalassemia alleles in Egypt using naturally or amplified created restriction sites and direct sequencing: a step in disease control. Hemoglobin.

[34] Omar A, Abdel Karim E, Gendy WE, Marzouk I, Wagdy M (2005). Molecular basis of beta-thalassemia in Alexandria. Egypt J Immunol..

[35] El-Gawhary S, El-Shafie S, Niazi M, Aziz M, El-Beshlawy A (2007). Study of beta-Thalassemia mutations using the polymerase chain reaction-amplification refractory mutation system and direct DNA sequencing techniques in a group of Egyptian Thalassemia patients. Hemoglobin..

[36] Elmezayen AD, Kotb SM, Sadek NA, Abdalla EM (2015). β-Globin Mutations in Egyptian Patients With β-Thalassemia. Lab Med..

[37] Huisman, T.H.J., Carver, M.F.H. & Baysal E. In Beta-thalassemia; nondeletional mutants. (Eds Huisman, T. H. J., Carver, M. F. H., Baysal, E., Marianne, F. H.). A syllabus of thalassemia mutations. Augusta, GA: The Sickle Cell Anemia Foundation. 1–309 (1997).

[38] Wood JC, Tyszka JM, Carson S, Nelson MD, Coates TD (2004). Myocardial iron loading in transfusion-dependent thalassemia and sickle cell disease. Blood.

[39] Eghbali A, Taherahmadi H, Shahbazi M, Bagheri B, Ebrahimi L (2014). Association between serum ferritin level, cardiac and hepatic T2-star MRI in patients with major β-thalassemia. Iran J Ped Hematol Oncol..

[40] Fragasso A (2011). Myocardial iron overload assessed by magnetic resonance imaging (MRI) T2* in multi-transfused patients with thalassemia and acquired anemias. Eur J Intern Med..

[41] Pennell DJ (2014). A 1-year randomized controlled trial of deferasirox versus deferoxamine for myocardial iron removal in beta-thalassemia major (CORDELIA). Blood..

[42] Verlhac S (2015). Liver iron overload assessment by MRI R2* relaxometry in highly transfused pediatric patients: an agreement and reproducibility study. Diagn Interv Imaging..

[43] Skordis N (2006). The impact of genotype on endocrine complications in thalassaemia major. Eur J Haematol..

[44] Yaman A (2013). Common complications in Beta thalasemia patients. Int. J Hematol Oncol.

[45] Winichagoon P, Fucharoen S, Chen P, Wasi P (2000). Genetic factors affecting clinical severity in beta-thalassemia syndromes. J Pediatr Hematol Oncol..

[46] Kattamis C (1982). The clinical phenotype of beta and delta beta thalassemias in Greece. Eur J Pediatr..

[47] Galanello R, Cao A (1998). Relationship between genotype and phenotype. Thalassemia intermedia. Ann N Y Acad Sci..

[48] Shamoon RP (2015). Molecular Basis of β-Thalassemia Intermedia in Erbil Province of Iraqi Kurdistan. Hemoglobin..

[49] Camaschella C (1995). Genetic interactions in thalassemia intermedia: analysis of beta-mutations, alpha-genotype, gamma-promoters, and beta-LCR hypersensitive sites 2 and 4 in Italian patients. Am J Hematol..

[50] Najafipour F (2008). Evaluation of endocrine disorders in patients with thalassemia major. Int J Endocrinol Metab..

[51] Naredi MN, Seth LC, Sharma CA (2011). Iron overload: A cause of primary amenorrhea. Medical Journal Armed Forces India..

[52] Jensen CE (1997). Incidence of endocrine complications and clinical disease severity related to genotype analysis and iron overload in patients with beta- thalassemia. Eur J Haematol..

[53] Classification and diagnosis of diabetes mellitus and other categories of glucose intolerance. *National Diabetes Data Group. Diabetes* **28**, 1039–1057 (1979). 10.2337/diab.28.12.1039 510803

[54] Cheng HL, Holowka S, Moineddin R, Odame I (2012). Liver iron overload assessment by T magnetic resonance imaging in pediatric patients: An accuracy and reproducibility study. American J Hematol..

